# Surgical retrieval of an embolized patent ductus arteriosus closure device in an infant weighing 1050 G

**DOI:** 10.1093/icvts/ivad162

**Published:** 2023-09-22

**Authors:** Arif Selcuk, Arvind Kumar Bishnoi, Joshua Kanter, Can Yerebakan

**Affiliations:** Division of Cardiac Surgery, Children's National Hospital, George Washington University School of Medicine and Health Sciences, Washington, DC, USA; Division of Cardiac Surgery, Children's National Hospital, George Washington University School of Medicine and Health Sciences, Washington, DC, USA; Division of Cardiology, Children's National Hospital, George Washington University School of Medicine and Health Sciences, Washington, DC, USA; Division of Cardiac Surgery, Children's National Hospital, George Washington University School of Medicine and Health Sciences, Washington, DC, USA

**Keywords:** Patent ductus arteriosus, Prematurity, Low birth weight, Cardiopulmonary bypass

## Abstract

The dislodgement of device during transcatheter procedure is a rare complication and the device can be retrieved by transcatheter techniques in most cases. In case of failed attempts, the surgery may be required and in haemodynamically unstable patients cardiopulmonary bypass (CPB) may be unavoidable. A case of surgical retrieving of patent ductus arteriosus (PDA) occlusion device (OD) from the right pulmonary artery (PA) in a 1050 g baby on CPB was presented. In literature, CPB use in babies weighing under 1 kg has been rarely reported. CPB support was performed securely in our case who is one of the tiniest patients operated on. CPB can be safe enough in the surgical approach of a complication of very low birth weight patient.

## INTRODUCTION

Interventional closure of haemodynamically significant PDA may be complicated. Following failed medical therapy, surgery has been long considered as a second choice. However, recently, the transcatheter closure option has been considered an alternative to surgery even for very low birth weight (VLBW, <1500 g) and premature infants. By increasing the number of patients who are treated with new-generation OD, surgery can be necessary for any device-related complications during or after implantation.

## MATERIALS AND METHODS

A case presentation of surgical retrieving of PDA OD in a 1050-g baby on CPB. The Children’s National Medical Center Institutional Review Board approved the study, and waiver of informed consent was obtained (IRB approval number and date: Pro00015566—07/01/2021).

## CASE PRESENTATION

A 38-day-old girl at 25.6 weeks gestation was referred for device retrieval into the right PA and surgical closure of a PDA. Her weight was 1050 g, and she had a large and haemodynamically significant PDA. After failed medical courses of PDA closure attempts the transcatheter device closure of PDA was planned. During the procedure, the OD migrated and lodged in the mid portion of the right PA. Further manoeuvres of catheter-based retrieval could not succeed because of the technical difficulty of catheter manipulation related to the size of patient and inappropriate stuck positioning of device. Transthoracic echocardiography and fluoroscopic guidance were used to determine the location of the device. The patient was then immediately transported to the operating room with CPB stand-by. Under general anaesthesia and through median sternotomy, the PA was approached. PDA was doubly ligated initially. The OD was palpable from the outside of right PA. However, the patient became desaturated as soon as right PA origin, and the distal right PA was snared for a device retrieval without CPB. It was decided to utilize CPB. After a brief CPB run of 21 min using aortic and right atrial cannulation, the OD was extracted from right PA following placement of snares on the main PA, right PA and left PA and through an incision on the main PA on beating heart (Video 1). The procedure was well tolerated. She was transferred to the cardiac intensive care unit (CICU) following uncomplicated weaning from CPB with open sternum. Delayed sternal closure was performed on the following day. No complications were observed. Her stay in CICU was uneventful and she was transferred to the neonatal intensive care unit on postoperative day 3.

## DISCUSSION

Presence of a PDA occurs in 39% of VLBW infants (1). Until 2019, the devices that were used for percutaneous PDA closure were not appropriate to be utilized for VLBW premature infants. Following the introduction of the Amplatzer Piccolo OD (Abbott Structural Heart, Plymouth, MN), the initial results are favourable. In a multicentre study of 645 PDA patients with 10% VLBW infants, the device embolization (2%), left PA stenosis (0.4%), descendant aorta stenosis (0.05%), cardiac perforation and death (0.01%) were observed (2).

According to a study on complications of Amplatzer Piccolo OD, the device embolization occurs into one of the PA branches, mostly left PA, more than into the systemic circulation and the embolization rate among infants ≤2 kg is reported 2%. Embolized devices into the pulmonary circulation can generally be retrieved with a transcatheter snare (>95%), but sometimes it may be necessary to proceed with surgery (3).

On the other hand, with faster weaning of respiratory support and lower morbidity rate, the transcatheter PDA closure in VLBW infants presents efficient and safer approach over surgical PDA closure (4). Under the guiding of recent reports (2, 3), the reliability and feasibility of transcatheter PDA closure in VLBW infants is well accepted. Our primarily choice for the closure of PDA in these patients is transcatheter approach.

As the number of implants is increasing, complications of transcatheter approach may be subject for surgical intervention. Mostly, an off-pump approach is convenient for device retrieval. CPB may become unavoidable in rare cases.

CPB use in babies weighing under 1 kg has been rarely reported (5). Nonetheless, in our case, CPB support was performed safely because of her cardiorespiratory instability during right PA control using snares.

## CONCLUSION

CPB can be safely used for device retrieval in VLBW infants in case of a rare complication of device dislodgement during transcatheter procedures.

## Data Availability

All relevant data are within the manuscript and its Supporting Information files.
